# Prenatal Intervention for Lower Urinary Tract Obstruction

**DOI:** 10.1100/tsw.2009.49

**Published:** 2009-05-29

**Authors:** David M. Kitchens, C. D. Anthony Herndon

**Affiliations:** University of Alabama at Birmingham, Birmingham, AL, USA

**Keywords:** fetal surgery, obstructive uropathy, oligohydramnios

## Abstract

Hydronephrosis is one of the most common abnormalities detected on routine prenatal ultrasounds, being noted in up to 1% of fetuses. Rarely, severe hydronephrosis coexists with oligohydramnios, which portends a poor prognosis. We review the most recent literature on the results of prenatal intervention in this setting. Presently, the first randomized controlled trial to address whether prenatal vesicoamniotic shunting improves survival and renal function is underway, and should address the value of prenatal intervention.

## INTRODUCTION

Routine screening ultrasounds are typically performed in the U.S. during the 18–20$^{th}$ week, gestational age. Fetal sonography has significantly changed the detection of genitourinary anomalies, which are generally able to be detected in the second trimester. It is estimated that about 1% of fetuses will have hydronephrosis. Significant hydronephrosis, manifested by bilateral hydronephrosis, a dilated bladder, and oligohydramnios, portends a poor prognosis for overall survival of the fetus[1]. Prenatal intervention to relieve lower urinary tract obstruction has been proposed if it appears that the life of the fetus is at imminent risk. These patients are clearly the most severely affected by obstructive uropathy, and it is important to realize that this situation rarely exists and careful consideration should be given prior to any proposed intervention[2].

## DECISION FOR INTERVENTION

Once it has been determined that second-trimester oligohydramnios or anhydramnios coexists with a dilated bladder and bilateral hydroureteronephrosis, a full evaluation of the fetus should be undertaken. A normal karyotype should be established via amniocentesis or chorionic villus sampling, and a full sonographic evaluation performed. At a minimum, if prenatal intervention is considered, the fetus should have documentation of no other systemic abnormalities, the absence of cysts in the renal parenchyma, and favorable urinary indices obtained via serial vesicocentesis. In an ideal setting, at least two urine samples should be obtained via vesicocentesis to document urinary indices. In general, urinary indices favoring a potential benefit in survival and overall renal function from prenatal intervention include sodium <100 mmol/L, chloride <90 mmol/L, calcium <8 mg/dL, osmolality <200 mmol/L, and β-2 microglobulin <6 mg/L[3]. The decision to intervene should be undertaken in the setting of a multidisciplinary approach, involving input from the Maternal Fetal Medicine specialists, pediatric nephrologists, and pediatric urologists. Informed consent should document the possible risks to the fetus, including premature delivery or fetal demise, the poor overall prognosis, the possibility of multiple procedures prior to delivery, the possibility of preterm labor, chorioamnionitis, spontaneous rupture of membranes, intestinal herniation, amniotic fluid leak, and the high likelihood of abnormal renal function. The option of termination of the pregnancy should also be addressed. Since renal function is not likely to be completely salvageable in this population, one of the main goals of intervention should be to achieve normalization of amniotic fluid levels to promote lung development. If lung development is mature enough to sustain life, then neonatal dialysis, if necessary, allows the possibility of early renal replacement therapy until a time when renal transplantation can safely be undertaken[4].

## RESULTS OF INTERVENTION

By far, the most common method of prenatal intervention is early delivery of the fetus, once it is felt that the lungs are mature enough to sustain life. In the most severe cases, the decision for invasive intervention is made in the second trimester when early delivery is not feasible. Several different methods of prenatal intervention, including placement of a vesicoamniotic shunt, creation of cutaneous ureterostomy, antegrade laser ablation of posterior urethral valves, antegrade hydroablation of posterior urethral valves, and antegrade passage of a guidewire, have been described to facilitate fetal bladder emptying due to lower urinary tract obstruction[3,5,6]. Documentation of the normalization of amniotic fluid levels is essential after intervention.

Most published reports consist of outcomes with placement of vesicoamniotic shunts. Selection of patients who might benefit from prenatal intervention was originally based on the work of Crombleholme and Johnson[see 7,8]. Patients with favorable urinary electrolyte profiles and the absence of renal cysts were felt to have the best outcomes from prenatal intervention. Recently, the groups from San Francisco, Toronto, and Philadelphia have published their results of patients who underwent prenatal intervention for severe second-trimester hydronephrosis with oligohydramnios. Only eight out of 14 (57%) survived prenatal intervention in Baskin's series of patients with posterior urethral valves; however, all eight patients were still alive with five out of eight (63%) of these patients having a glomerular filtration rate (GFR) <70 mL/min/1.73 $m^2$ at a mean follow-up of 11.6 years[6]. Interestingly, all patients in Baskin's cohort had favorable prenatal urinary indices, but this did not appear to predict postnatal renal function. In the Toronto series of patients with vesicoamniotic shunts, McLorie et al. report that six out of nine (67%) patients initially survived following intervention, with five out of six (83%) still alive and three out of six (50%) having a GFR <70 mL/min/1.73 $m^2$ at a mean follow-up of just under 4 years[9]. Unlike Baskin's cohort, though, patients in this series had diagnoses of posterior urethral valves, urethral atresia, and prune belly syndrome. In 2005, Biard et al. reported survival rates up to 91% at 1 year of age following placement of vesicoamniotic shunts[3]. They also noted that patients with posterior urethral valves tended to have more favorable renal function than those with urethral atresia or prune belly syndrome. Recently, we presented data on short-term outcomes with prenatal intervention in patients with poor urinary electrolyte parameters[10]. We were able to obtain a 30% survival in this population at a median follow-up of just under 2 years. Obviously it is difficult to compare patients in different series because dissimilar parameters were used to identify those who met criteria for shunting and survival end points are not always noted[3,6,7,8,9,10,11,12,13].

## CONCLUSIONS

Presently, prenatal intervention for significant lower urinary tract obstruction is controversial, at best, and has been limited to a handful of centers in North America[3,6,8,9,10,13]. At this time, definitive correction of the underlying pathology accounting for the obstruction cannot be achieved until after delivery. Based on the lack of randomized controlled trials in the literature, it is difficult to determine whether prenatal intervention for lower urinary tract obstruction is truly beneficial to eventual renal function or overall survival. In Europe, however, a multicenter, randomized, controlled trial (**P**ercutaneous shunting for **L**ower **U**rinary **T**ract **O**bstruction randomized controlled trial [PLUTO]) that addresses whether prenatal vesicoamniotic shunting improves perinatal mortality and eventual renal function is currently being conducted[14]. As more information is published on the results of prenatal intervention, we should obtain a better grasp on exactly which patients will benefit from this intervention.
